# A Stevedore's Protein Knot

**DOI:** 10.1371/journal.pcbi.1000731

**Published:** 2010-04-01

**Authors:** Daniel Bölinger, Joanna I. Sułkowska, Hsiao-Ping Hsu, Leonid A. Mirny, Mehran Kardar, José N. Onuchic, Peter Virnau

**Affiliations:** 1 Department of Physics, Johannes Gutenberg-Universität Mainz, Mainz, Germany; 2 CTBP, University of California San Diego, San Diego, California, United States of America; 3 Harvard-MIT Division of Health Sciences and Technology, Cambridge, Massachusetts, United States of America; 4 Department of Physics, Massachusetts Institute of Technology, Cambridge, Massachusetts, United States of America; National Institute for Medical Research, United Kingdom

## Abstract

Protein knots, mostly regarded as intriguing oddities, are gradually being recognized as significant structural motifs. Seven distinctly knotted folds have already been identified. It is by and large unclear how these exceptional structures actually fold, and only recently, experiments and simulations have begun to shed some light on this issue. In checking the new protein structures submitted to the Protein Data Bank, we encountered the most complex and the smallest knots to date: A recently uncovered α-haloacid dehalogenase structure contains a knot with six crossings, a so-called Stevedore knot, in a projection onto a plane. The smallest protein knot is present in an as yet unclassified protein fragment that consists of only 92 amino acids. The topological complexity of the Stevedore knot presents a puzzle as to how it could possibly fold. To unravel this enigma, we performed folding simulations with a structure-based coarse-grained model and uncovered a possible mechanism by which the knot forms in a single loop flip.

## Author Summary

Knots are ubiquitous in many aspects of our life, but remain elusive in proteins. The multitude of protein structures archived in the Protein Data Bank can be grouped into several hundred patterns, but only a handful are folded into knots. Combing through the recently added structures we found several novel knotted proteins. A microbial enzyme that catalyzes the breakdown of pollutants is the most complex protein knot encountered so far (similar to a knot used by stevedores for lifting cargo). The smallest knotted protein on the other hand consists of only 92 amino acids. The existence of these complex motifs demonstrates that the ability of self assembly goes far beyond normal expectations. Aided by computer simulations we present evidence which suggests that the Stevedore protein knot, despite its topological complexity, may actually form in a single flipping movement.

## Introduction

In the last decade, our knowledge about structure and characteristics of proteins has considerably expanded. The ability of proteins of small and medium size to fold into native structures is attributed to a minimally frustrated free energy landscape, which allows for fast and robust folding [1],[2]. In recent years, however, a new class of proteins with knotted topologies emerged [3],[4],[5],[6],[7] that broadened the scope of possible folding landscapes.

Not withstanding our daily experiences with shoelaces and cables, knots are mathematically only properly defined in closed loops, and not on open strings. In proteins, however, this issue can be resolved by connecting the termini (which are usually located on the surface) by an external loop [3],[4],[7]. This approach actually corresponds to a more practical definition of knottedness in which we demand that a knot remains on a string and tightens when we pull on both ends. After such closure, mathematical algorithms like the Alexander polynomial [8] can be employed to determine the type of knot (a topological invariant). Knots are usually classified according to the minimum number of crossings in a projection onto a plane. Most knotted proteins discovered to date are quite simple. Out of the seven distinctly knotted folds discovered to date (see Table 1), four are simple trefoil knots (3_1_) with 3 crossings, two are figure-eight knots (4_1_) with 4 crossings, and only one fold is made up of five crossings (5_2_). Most of the knots in protein structures, however, were initially undetected from their structures since finding them by visual inspection is fairly hard, requiring a computational approach.

**Table 1:** Proteins with knotted backbones.

protein family	pdb	chain start-stop	knot type	knotted core
RNA methytransferase (**α/β** knot)	1ns5	1–153	3_1_	69–121
Carbonic anhydrase	1lug	2–260	3_1_	31–257
SAM synthetase	1fug	1–383	3_1_	33–260
Transcarbamylase fold	1js1	1–324	3_1_	169–267
*Zinc-finger fold*	2k0a	−1–107	3_1_	18–78
*Ribbon-helix-helix superfamily*	2efv	6–87	3_1_	19–66
CII Ketol-acid reductoisomerase	1yve	83–595	4_1_	321–533
Chromophore binding domain*	1ztu	5–325	4_1_ *	41–298
Ubiquitin Hydrolase	2etl	1–223	5_2_	10–216
**α** *-haloacid dehalogenase I*	3bjx	−14–296	6_1_	71–268

For each fold an example pdb code is given. Chain start-stop refers to the first and the last amino acid, which are resolved in the structure. The knotted core is the minimum configuration which stays knotted after a series of deletions from either terminus as given by our web server [37]. This “knot size” is determined by an automated procedure [7],[37], and results should only be regarded as a guideline.

*: There are several missing (unresolved) amino acids in 1ztu – the complete structure will likely contain a figure-eight knot. Slipknots are not listed in this table, which (of course) also contain knots in their backbone.

Even though some pioneering experiments [9],[10],[11],[12],[13] have began to shed some light on how these peculiar structures fold and unfold, still little is known about the exact mechanisms involved. Recently, this subject was addressed with simulations of structure-based coarse-grained models [14],[15] that suggested for the first time potential folding mechanisms and unfolding pathways [14],[15],[16],[17],[18],[19] for knotted proteins. It has been suggested that folding of knotted proteins may proceed through an unfolded but knotted intermediate by simulations which include non-native contacts [14], or by formation of slipknot conformations [15] (segments containing a knot which disappears when protein as a whole is considered) in conjunction with partial folding and refolding (backtracking) events [20]. The slipknot conformations allow the protein to overcome topological barriers in the free energy landscape which might otherwise lead to kinetic traps [21],[22],[23]. In a more general context, it is also intriguing to ask if the folding of complex knots can be reconciled with the folding funnel hypothesis [1],[2] or nucleation mechanisms [24].

In this paper we present the most complex and also the smallest, knotted proteins known to date. To shed some light on potential folding routes of the former, we undertook molecular dynamics simulations with a coarse-grained model which only includes native contacts. Even though it is intrinsically difficult to fold such a large protein with a simple structure-based model, a small fraction of our trajectories (6 out 1000) folded into the knotted native state. Based on these simulations we propose a new mechanism by which this complex protein knot may fold in a single flipping movement. The proposed mechanism differs from mechanisms suggested before as it involves the flipping of a large loop over a mostly folded structure rather than folding via mostly unstructured knotted intermediates [14].

## Results

### Analysis of the protein data bank

#### The most complex protein

**Figure 1:**
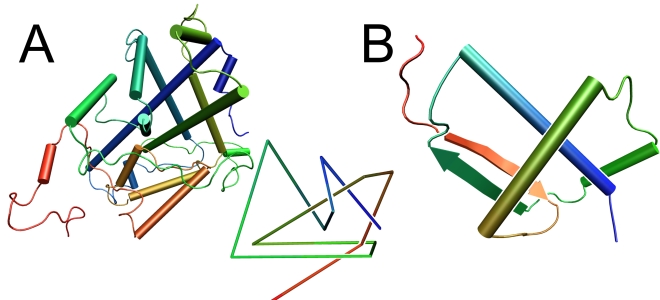
Protein crystal structure. a: Crystal structure of α-haloacid dehalogenase DehI (PDB code 3bjx). The chain is composed of two homologous regions that form a pseudodimer and are connected by a proline-rich arc. The insert shows a reduced schematic representation of the protein. b: Crystal structure of the smallest knot discovered in an uncharacterized protein (PDB code 2efv). Both pictures were prepared with VMD [38].

By systematically analyzing structures submitted to the PDB [25] up to August 2009, we discovered an imposing knot in an **α**-haloacid dehalogenase DehI [26]. DehI is a member of a large family of dehalogenases, microbial enzymes that catalyze the breakdown of organic pollutants by cleaving the carbon-halogen bond, and are of interest for bioremediation. The homodimer DehI shares no sequence or structural similarity to other dehalogenases and has a novel fold. A reduced representation of the protein in fig. 1a reveals six crossings belonging to a so-called Stevedore knot (6_1_) – a type of stopper knot used by stevedores to prevent large blocks from running through the line while raising cargo. The resulting knot is quite deep and will not vanish if a few amino acids are cut from either side. In fact one could cleave more than 20 amino acids from the C-terminus and around 65 residues from the N-terminus without destroying the knotted topology. The DehI monomer consists of two regions (∼130 a.a. each) that share about 20% sequence identity (Needleman-Wunsch sequence alignment with Blosum62 matrix, gap opening = 7, gap extension = 1), have very similar structures [26], and are likely to result from a tandem sequence duplication. The structure of each fragment is unknotted, but their assembly into the whole DehI structure creates a knot. The two regions are connected by a proline-rich loop that goes around the protein forming a large arc (fig. 1a).

#### The smallest knotted protein

While DehI constitutes the most complex knot found so far, another protein was detected by our algorithm as having the smallest known knot. The backbone of an uncharacterized protein MJ0366 from *M.jannaschii*, solved by Structural Genomics/Proteomics Initiative [27] has only 92 amino acids (of which 82 are resolved in the pdb structure, 2EFV) and forms a novel fold with a trefoil knot (fig. 1b). A visual inspection reveals that around 10 amino acids (including unstructured amino acids missing in the pdb-file) can be cleaved from the C-terminus and around 20 amino acids from the N-terminus before the knot disappears. During the review process we learned that the knot in MJ0366 was also discovered independently by Alexey Murzin soon after the structure was released in August 2007 (MK first presented 2efv and 3bjx in a seminar at MIT in May 2008). It is also listed in the current version of SCOP [28] (1.75, June 2009). The protein belongs to the ribbon-helix-helix (RHH) superfamily of DNA-binding proteins and is the first knotted protein of its kind. The subunit is similar to the dimeric folds of typical RHH proteins, like the Arc repressor, and likely resulted from a gene duplication/fusion event. Two RHH motifs are connected with a linker and the specific locations of N- and C- termini in the dimeric RHH folds suggest that the addition of the linker may have created this knot. Note that gene duplication/fusion events may have contributed to the origin of knotted proteins, too.

We also discovered two additional knotted DNA binding proteins. VirC2 (virulence protein from a plasmid of *Agrobacteria*) [29] is also made up of a duplicated RHH motif and folds into a trefoil (as noted in SCOP 1.75) which is almost identical to the one observed in MJ0366. Intriguingly, the two share only 9% of their sequence. Finally, we noted a knotted zinc-finger, which was already discussed in Ref. [30].

### Folding simulations of DehI

It is difficult to imagine how proteins can actually fold into topologically elaborate structures like the 6_1_ knot displayed in fig. 1a. Complex knots, however, are not necessarily difficult to tie. There are actually quite a few rather complicated knots, including the Stevedore knot in DehI, which can be transformed into unknots by removing a single crossing. Likewise, these knots can typically be formed in a single movement which simplifies the folding of these peculiar structures considerably. Recently, Taylor [31] predicted that complex protein knots discovered in the future will most likely belong to this class which is corroborated by the discovery of the Stevedore knot in DehI. As indicated in [31], knots of arbitrary complexity can be obtained by twisting a loop in a string over and over again before threading one end through the loop. Even though this way of creating knots may appear as an attractive protein folding scenario due to its simplicity, our results suggest a somewhat different potential mechanism, which is able to reduce topological constraints and fold DehI in a single movement.

Two loops are crucial for the formation of the 6_1_ knot in DehI: a smaller loop which we call S-loop containing amino acids 64 to 135 and a slightly bigger loop termed B-loop ranging from amino acid 135 to 234. Note that the latter includes the proline rich unstructured segment mentioned earlier. The analysis of the crystallographic B-factor (see fig. S1) reveals that the center of the S-loop, the beginning and the end of the B-loop, as well as the unstructured proline rich segment, are particularly mobile. In addition, a very mobile unstructured segment around amino acid 240 provides additional flexibility to the C-terminus. Note that if the B-loop is flipped over to the other side of the protein, the Stevedore knot disentangles in a single step.

In an attempt to elucidate the folding route of DehI, we undertook molecular dynamics simulations with a coarse-grained structure based Go-model [1],[32],[33] of DehI which does not include non-native interactions. With this model we were able to fold six trajectories (out of 1000) into the 6_1_ knot (with more than 90% of native contacts). We emphasize that this number should not be associated with experimental folding rates. Folding large knotted proteins with a generic structure-based model without non-native interactions is extremely difficult as the protein has to undergo a series of twists and threading movements in correct order while collapsing. As demonstrated in Ref. [14] the addition of non-native interactions will increase the folding rate substantially, however, at the cost of introducing a bias. There is also a strong dependence of successful folding events on protein size. For example, in Ref. [15] a rather simple and short trefoil knot in an RNA methyltransferase, folded successfully in only 2% of all cases with the same underlying model. On the other hand we succeeded in folding 2efv with 100% success rate [34]. For comparison the number of amino acids in 2efv is roughly two times smaller than the number of amino acids in the methyltransferase, which again is roughly two times smaller than the number of amino acids in the dehalogenase. While acknowledging such limitations of coarse-grained models, we are still confident in deducing a potential folding pathway from the analysis of the successful trajectories, in particular because all six trajectories are very similar.

**Figure 2:**
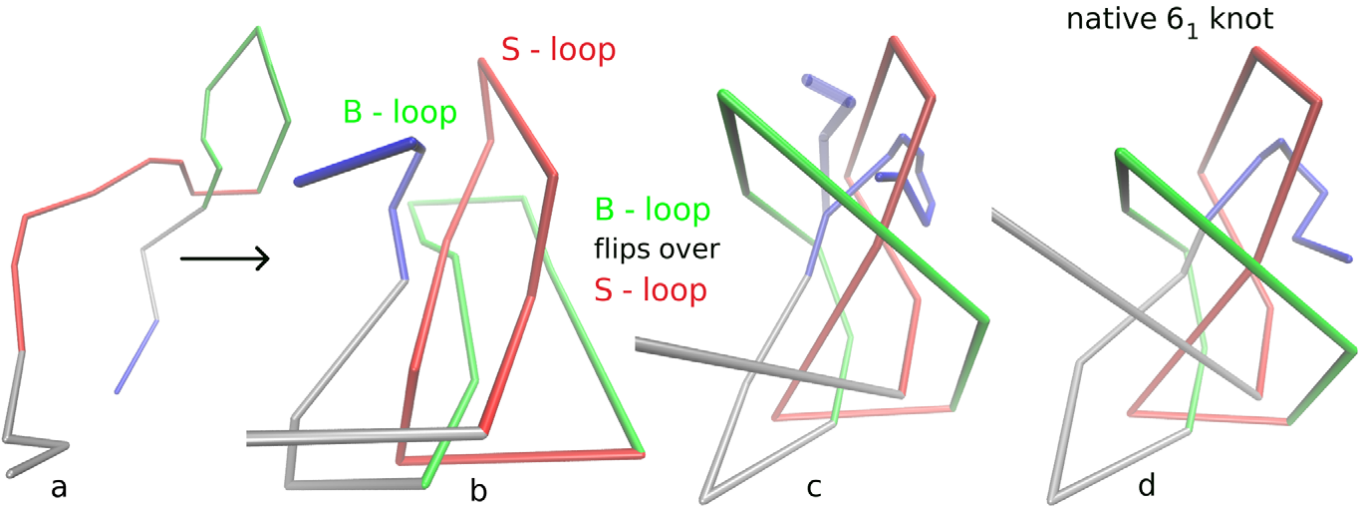
Snapshots taken from a folding trajectory of DehI (0→6_1_). The S-loop (amino acids 64 to 135) is colored red, the B-loop (amino acids 135 to 234) green and the C-terminus blue. a: B- and S-loop form in the beginning by twists of the partially unfolded protein. b: B- and S-loop align. c: the S-loop twists once again, the C-terminus threads through the S-loop (in slipknot conformation) and the B-loop flips over the S-loop. In the alternative folding scenario (0→4_1_→6_1_), the B-loop flips over the (twisted) S-loop before the C-terminus (indicated in light blue) threads through the S-loop (4_1_), shortly after the C-terminus threads through the S-loop in slipknot conformation. d: Native state without slipknotted C-terminus.

Fig. 2 shows an actual folding trajectory. The S-loop is colored red, the B-loop green and the C-terminus blue. Two very similar potential folding routes were observed in our simulations. In both routes, the two loops form in the beginning by twists (fig. 2a) of the partially unfolded protein such that B- and S-loop are aligned (fig. 2b). In the first route, the C-terminus is threaded through the S-loop (which needs to twist once again – fig. 2c) before the B-loop flips over the S-loop. In the second route the steps are interchanged: the B-loop flips over the S-loop and the C-terminus (shaded in light blue in fig. 2c). A figure-eight (4_1_) knot forms as a result before the C-terminus manages to thread through the S-loop to reach the native state. In both cases, the C-terminus moves through the S-loop via a slipknot conformation (fig. 2c). Note that loop flipping and threading are typically accomplished with backtracking events [20] for topologically frustrated proteins [21]. Similar conformational changes during folding mechanisms have been observed in other topologically non-trivial structures. The rotation of a proline rich loop was also observed in a big slipknotted protein, Thymidine Kinase [15]. Slipknot intermediates appear in the folding mechanism for the trefoil knot in Methyltransferase [15] as well.

Unfortunately, the size and complexity of the protein does not allow us to study the full thermodynamic process and reconstruct the free energy profile along a reaction coordinate. However, kinetic data allow us to distinguish some characteristic times from which we can deduce a likely folding mechanism.

**Figure 3:**
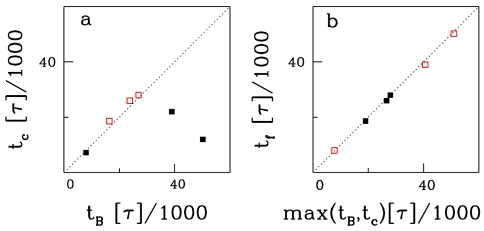
Folding times. a: t_B_ time (in units of MD-time steps) of flipping the B-loop over the S-loop versus time of threading the C-terminal through the S-loop (t_c_). Solid symbols are trajectories associated with route I (0→6_1_), open symbols are trajectories associated with route II (0→4_1_→6_1_). b: t_f_ – folding time (at which 90% of native contacts have been established) versus maximum of t_B_ or t_c_.

In fig. 3 we investigate the rate-limiting step in the folding of the Stevedore knot. On the left panel, we plot the time it takes to thread the C-terminus through the S-loop (t_c_) against the time it takes to flip the B-loop over the S-loop. Solid symbols are trajectories associated with route I (0→6_1_), and open symbols are trajectories associated with route II (0→4_1_→6_1_). In the first pathway, the flipping of the B-loop takes longer than the threading of the C-terminus in two out of three cases. In the second pathway (and the third trajectory associated with route I), the threading of the C-terminus through the S-loop occurs shortly after the flipping of the B-loop. In both scenarios, the flipping of the B-loop over the S-loop is the rate-limiting step. Once this is achieved, the protein is essentially folded (fig. 3b). The flipping of the B-loop can therefore be associated with an entropic barrier in the folding free energy. From an analysis of the order at which contacts occur (fig. S2) it is possible to deduce the occurrence of a first small barrier, which is associated with the formation and twisting of B- and S-loop before the B-loop flips. Hence, we believe a three-state folding scenario is more likely than a two-state scenario.

In order to study the unfolding pathway, we raised the temperature above the folding temperature. Even though some native contacts are lost at higher temperatures, the global mechanism is by and large reversed as compared to the folding routes (see fig. S3).

To check how topological complexity restricts the free energy landscape the protein topology was changed from 6_1_ to 4_1_ (by eliminating a crossing, as previously performed with a different protein in Ref. [35]). This slight modification increases the folding ability of DehI substantially to 11%, suggesting that complexity of the knot is an important parameter in determining the foldability of a protein.

## Discussion

Our analysis of the Protein Data Bank revealed the most complex protein knot in **α**-haloacid Dehalogenase DehI and the shortest (so far unclassified) knotted protein known to date. This discovery underscores that knots in the backbone of proteins are significant structural motifs that appear at different levels of protein complexity and might offer new insight in the understanding of protein folding mechanisms. The finding of the smallest knotted protein (which is almost half the size of all previously known protein knots) may eventually enable us to study the folding of knotted proteins with more sophisticated all-atom simulations.

We investigated the folding route of the most topologically complex protein knot with molecular dynamics simulations of a structure-based model. The analysis of successful folding trajectories suggests that the Stevedore (6_1_) knot in DehI folds via a simple mechanism: a large twisted loop in the protein flips over another previously twisted loop, thus essentially creating the six-fold knot in a single movement. Thus, the topological complexity of the Stevedore knot in DehI can be overcome and explained in the context of classical theories of protein folding [1],[2],[36]. The flipping of a loop over a mostly folded structure constitutes a new scenario in the folding of knotted proteins which differs, e.g., from the folding of knots via partially unstructured knotted intermediates [14]. Our mechanism also includes previously observed elements like the threading of slipknot conformations through loops [15]. These mechanisms can be essential for folding into topologically challenging structures and provide a general framework for the understanding of knotted proteins.

## Methods

### Knot topology

The programs used to detect knots are identical to those used in our previous work [7]. To determine whether or not a structure is knotted, we reduce the protein to its backbone, and draw two lines outward starting at the termini in the direction of the connection line between the center of mass of the backbone and the respective ends. The knot type is determined by computing the Alexander polynomial, which is also implemented on our protein knot detection server (http://knots.mit.edu.) [37]. For a detailed discussion of our methods, the reader is referred to Ref. [7].

### Molecular dynamics simulations

Note that this class of structure based models was not created with protein knots in mind and is very prone to fold into topologically frustrated states. Even though Go-models can be adapted to enhance the formation of knots [14] we refrained from this approach because we did not want to impose any bias. We applied a structure based coarse-grained model with only native contacts [32],[33]. In total we folded 1000 trajectories of DehI at temperature T = 0.48 out of which 6 folded into a 6_1_ knot. Furthermore, we observed 737 unknotted conformations, 85 trefoil (3_1_), 167 figure-eight (4_1_) and five 5_2_ knots. Higher and lower temperatures resulted in a lower rate of 6_1_ formation. After the structure was simplified to a figure-eight knot, 11% of all configurations folded into the native state (with more than 95% native contacts.)

## Supporting Information

Figure S1 Structural elements of DehI and B-factors
(0.18 MB PDF)

Figure S2 Order of contact formation for the folding of DehI
(0.10 MB PDF)

Figure S3 Unfolding routes which lead to unknotted conformations
(0.06 MB PDF)

## References

[1] Bryngelson JD, Onuchic JN, Socci ND, Wolynes PG (1995). Funnels, Pathways, and the Energy Landscape of Protein-Folding - a Synthesis.. Proteins Struct Funct Genet.

[2] Leopold PE, Montal M, Onuchic JN (1992). Protein Folding Funnels - a Kinetic Approach to the Sequence Structure Relationship.. PNAS.

[3] Mansfield ML (1994). Are there knots in proteins?. Nature Struct Biol.

[4] Mansfield ML (1997). Fit to be tied.. Nature Struct Biol.

[5] Taylor WR (2000). A deeply knotted protein structure and how it might fold.. Nature.

[6] Lua RC, Grosberg AY (2006). Statistics of knots, geometry of conformations, and evolution of proteins.. PLoS Comput Biol.

[7] Virnau P, L AM, Kardar M (2006). Intricate Knots in Proteins: Function and Evolution.. PLoS Comput Biol.

[8] Livingston C (1993). Knot theory..

[9] Mallam AL, Jackson SE (2005). Folding studies on a knotted protein.. J Mol Biol.

[10] Mallam AL, Jackson SE (2006). Probing Nature's Knots: The Folding Pathway of a Knotted Homodimeric Protein.. J Mol Biol.

[11] Mallam AL, Jackson SE (2007). A comparison of the folding of two knotted proteins: YbeA and YibK.. J Mol Biol.

[12] Mallam AL, Onuoha SC, Grossmann JG, Jackson SE (2008). Knotted fusion proteins reveal unexpected possibilities in protein folding.. Mol Cell.

[13] Bornschlogl T, Anstrom DM, Mey E, Dzubiella J, Rief M (2009). Tightening the Knot in Phytochrome by Single-Molecule Atomic Force Microscopy.. Biophys J.

[14] Wallin S, Zeldovich KB, Shakhnovich EI (2007). The folding mechanics of a knotted protein.. J Mol Biol.

[15] Sulkowska JI, Sulkowski P, Onuchic JN (2009). Dodging the crisis of folding proteins with knots.. PNAS.

[16] Sulkowska JI, Sulkowski P, Szymczak P, Cieplak M (2008). Tightening of knots in proteins.. Phys Rev Lett.

[17] Huang L, Makarov DE (2008). Translocation of a knotted polypeptide through a pore.. J Chem Phys.

[18] Dzubiella J (2009). Sequence-Specific Size, Structure, and Stability of Tight Protein Knots.. Biophys J.

[19] Sulkowska JI, Sulkowski P, Onuchic JN (2009). Jamming proteins with slipknots and their free energy landscape.. Phys Rev Lett: 268103.

[20] Gosavi S, Whitford PC, Jennings PA, Onuchic JN (2008). Extracting function from a beta-trefoil folding motif (vol 105, pg 10384, 2008).. PNAS.

[21] Norcross TS, Yeates TO (2006). A framework for describing topological frustration in models of protein folding.. J Mol Biol.

[22] Yeates T, Norcross TS, King NP (2007). Knotted and topologically complex proteins as models for studying folding and stability.. Curr Opin Chem Biol.

[23] King NP, Yeates EO, Yeates TO (2007). Identification of rare slipknots in proteins and their implications for stability and folding.. J Mol Biol.

[24] Fersht AR (2008). From the first protein structures to our current knowledge of protein folding: delights and scepticisms.. Nat Rev Mol Cell Biol.

[25] Berman HM, Westbrook J, Feng Z, Gilliland G, Bhat TN (2000). The Protein Data Bank.. Nucleic Acids Res.

[26] Schmidberger JW, Wilce JA, Weightman AJ, Whisstock JC, Wilce MCJ (2008). The crystal structure of Dehl reveals a new alpha-haloacid dehalogenase fold and active-site mechanism.. J Mol Biol.

[27] Kumarevel TS, Karthe P, Kuramitsu S, Yokoyama S (to be published) Crystal Structure of a Hypothetical Protein(MJ0366) from Methanocaldococcus jannaschii..

[28] Murzin AG, Brenner SE, Hubbard T, Chothia C (1995). Scop - a Structural Classification of Proteins Database for the Investigation of Sequences and Structures.. J Mol Biol.

[29] Lu J, Glover JNM (to be published) Crystal structure of plasmid pTiC58 VirC2.

[30] van Roon AMM, Loening NM, Obayashi E, Yang JC, Newman AJ (2008). Solution structure of the U2 snRNP protein Rds3p reveals a knotted zinc-finger motif.. PNAS.

[31] Taylor WR (2007). Protein knots and fold complexity: Some new twists.. Comput Biol Chem.

[32] Cieplak M, Hoang TX (2003). Universality classes in folding times of proteins.. Biophys J.

[33] Clementi C, Nymeyer H, Onuchic JN (2000). Topological and energetic factors: What determines the structural details of the transition state ensemble and “en-route” intermediates for protein folding? An investigation for small globular proteins.. J Mol Biol.

[34] Noel JK, Sulkowska JI, Onuchic JN (2010). in preparation..

[35] Sulkowska JI, Sulkowski P, Szymczak P, Cieplak M (2008). Stabilizing effect of knots on proteins.. PNAS.

[36] Mirny LA, Shakhnovich EI (1996). How to derive a protein folding potential? A new approach to an old problem.. J Mol Biol.

[37] Kolesov G, Virnau P, Kardar M, Mirny LA (2007). Protein knot server: detection of knots in protein structures.. Nucleic Acids Res.

[38] Humphrey W, Dalke A, Schulten K (1996). VMD: Visual molecular dynamics.. J Mol Graphics.

