# Supplementary material for: A Stevedore's Protein Knot
Source: PLoS Comput Biol. 2010 Apr 1;6(4):e1000731. doi: 10.1371/journal.pcbi.1000731 (PMC2848546; doi:10.1371/journal.pcbi.1000731)
Supplement: Figure S1 — Structural elements of DehI and B-factors (0.18 MB PDF) [file pcbi.1000731.s001.pdf]

Supplementary Material

Daniel Bolinger, Joanna I. Sułkowska, Hsiao-Ping Hsu, Leonid A. Mirny, Mehran Kardar, Jose N. Onuchic and Peter Virnau

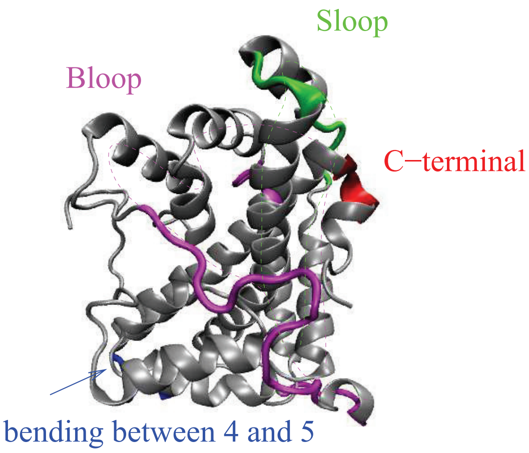

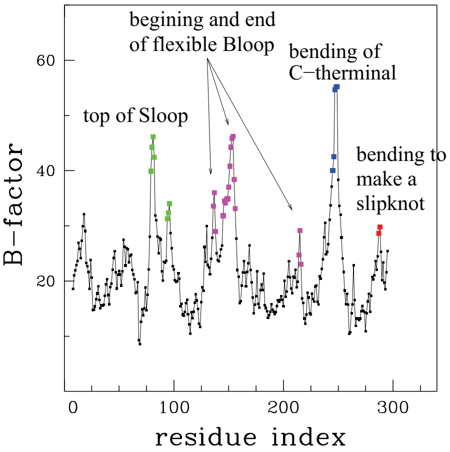

Figure 1: Structural elements of DehI and B-factors.
Left, cartoon representation of DehI. Colors highlight the 5 major structural sections of the protein 1. amino acids 1-64 - several α-helices, 2. amino acids 64-135: S-loop - two long α-helices, 3. amino acids 135-234: B-loop 3 α-helices connected by a proline-rich unstructured part of the backbone, 4. amino acids 234-252: segment of the backbone which crosses the B-loop, 5. Long α-helix which crosses B-loop and S-loop. Right, mean square fluctuations of individual residues presented as B factor.
