# Supplementary material for: A Stevedore's Protein Knot
Source: PLoS Comput Biol. 2010 Apr 1;6(4):e1000731. doi: 10.1371/journal.pcbi.1000731 (PMC2848546; doi:10.1371/journal.pcbi.1000731)
Supplement: Figure S2 — Order of contact formation for the folding of DehI (0.10 MB PDF) [file pcbi.1000731.s002.pdf]

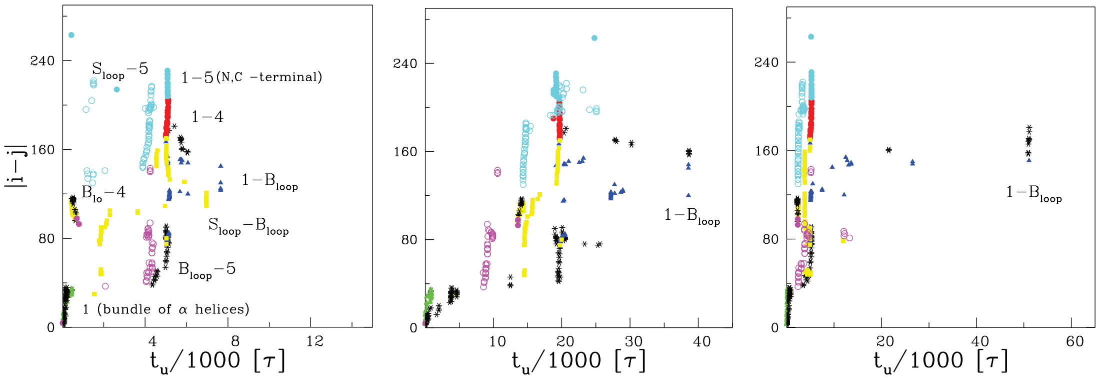

Figure 2: Order of contact formation for the folding of DehI. The time, t_u at which a contact is established for the first time, $|j-i|$ is the distance in sequence between two amino acids which have established contact. The left panel corresponds to the trajectory (0-6_1) which is shown in Figure S2 (main text), the middle panel corresponds to a trajectory belonging to pathway (0-4_1-6_1) and the right panel to another (0-6_1) trajectory. Formation of contacts between S-loop and B-loop are denoted as yellow squares. In this case high values for $|j-i|$ correspond to the formation of the B-loop and the twist of the S-loop before the flipping of the B-loop. Regions 1-5 are the same as in Figure S1. Solid magenta circles denote contacts between the B-loop and region 4, open magenta circles denote contacts between the B-loop and region 5, Green stars denote contacts between B-loop and region 1. All these contacts are responsible for the formation of the B-loop. Black stars with low values for $|j-i|$, open cyan circles denoting contacts between the S-loop and region 5 are all involved in the formation of the S-loop. Yellow squares with $|j-i|$ around 100, correspond to the formation of contacts between the major two loops (the flip of the B-loop over the S-loop). Blue triangles (1-B-loop) denote the final straightening of the C-terminus and conclude the formation of the 6_1 knot. The same notation is used for the middle and the right panel.
