# Supplementary material for: A Stevedore's Protein Knot
Source: PLoS Comput Biol. 2010 Apr 1;6(4):e1000731. doi: 10.1371/journal.pcbi.1000731 (PMC2848546; doi:10.1371/journal.pcbi.1000731)
Supplement: Figure S3 — Unfolding routes which lead to unknotted conformations (0.06 MB PDF) [file pcbi.1000731.s003.pdf]

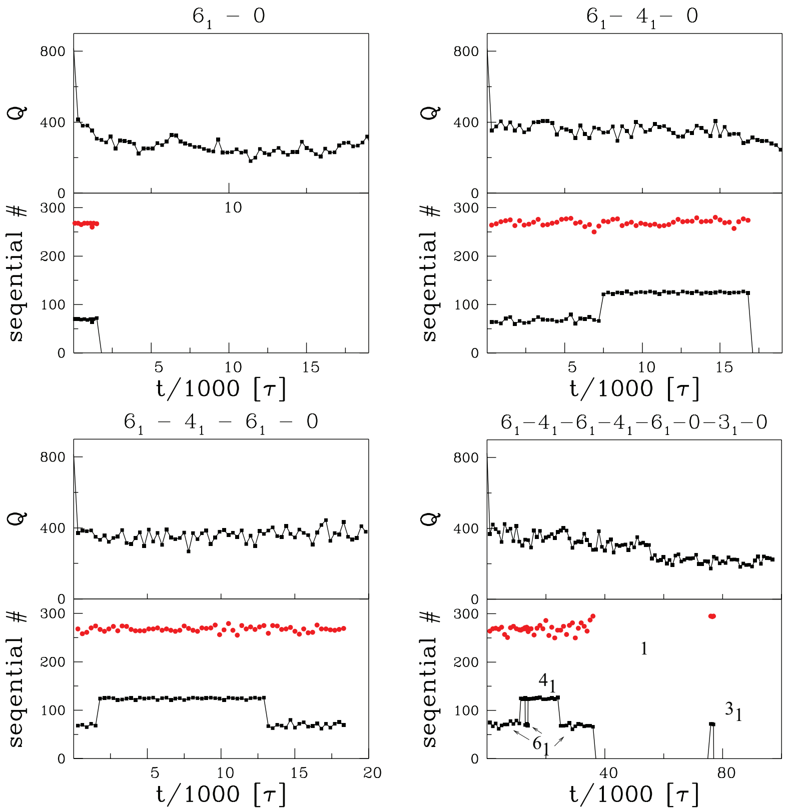

Figure 3: Unfolding routes which lead to unknotted conformations.

Top panels represent the Q values for a given time. The bottom panels display the position of the knot ends and the knot type.
